# Overcoming Challenges in the Commercialization of Biopolymers: From Research to Applications—A Review

**DOI:** 10.3390/polym16243498

**Published:** 2024-12-16

**Authors:** Simon Schick, Julia Heindel, Robert Groten, Gunnar H. Seide

**Affiliations:** 1 Aachen-Maastricht Institute for Biobased Materials (AMIBM), Faculty of Science and Engineering, Maastricht University, Brightlands Chemelot Campus, Urmonderbaan 22, 6167 RD Geleen, The Netherlands; s.schick@maastrichtunversity.nl; 2 Department of Business Administration, University of Applied Sciences Munich, Lothstrasse 34, 80335 Munich, Germany; 3 Department of Textile and Clothing Technology, Niederrhein University of Applied Sciences, Campus Mönchengladbach, Webschulstrasse 31, 41065 Mönchengladbach, Germany

**Keywords:** product development, degradation by design, biopolymers, degradability

## Abstract

Biopolymers are promising sustainable alternatives to petrochemical polymers, but the recent increase in published research articles has not translated into marketable products. Here, we discuss barriers to market entry by exploring application-specific, ecological, and economic aspects, such as the utilization of biodegradable polymers to mitigate the accumulation of microplastics. We summarize previous studies revealing how fiber surface properties and the dwell time during fiber spinning affect degradability. We show how biopolymers can be processed on existing machines and how degradability can be tailored by changing process parameters. This novel approach, known as degradation by design, will allow us to rethink product development and ensure that biopolymers are not only able to replace petrochemical polymers but also reduce the environmental harm they cause.

## 1. Introduction

### 1.1. Background

Polymers play a major role in numerous aspects of our daily lives, and it is difficult to envision a world without them [1]. In 2020, the world produced 367 metric tons of polymers, ~40% of which were used in packaging [2]. Most petrochemical polymers degrade extremely slowly in the environment and, thus, accumulate as macroplastics and microplastics [3,4], the latter ultimately finding their way into the human food chain [5]. The proper disposal and recycling of waste polymers has numerous benefits, including the creation of jobs, lower energy consumption [6], and, thus, a smaller carbon footprint for new products and lower raw material costs by reducing the need for virgin materials. Less waste ends up in landfills and incinerators or discarded in the environment [7]. 

Despite the benefits of recycling, less than 10% of polymers are reused [8]. The remaining 90% can be designated as managed or unmanaged waste. Jurisdictions such as the European Union (EU), the United States, Japan, South Korea, and Canada are among the best for waste stream management, with less than 2% designated as unmanaged [9]. In contrast, the highest proportions of unmanaged waste are found in Vietnam (86% of 5.3 million tons), India (85% of 16.7 million tons), and Indonesia (81% of 5.9 million tons) [9]. China is the largest contributor to unmanaged waste (16.96 million tons) [9]. Unmanaged waste is found predominantly as land waste and, to a smaller extent, sea waste [9]. Due to this mismanagement, material lost from the cycle must be replaced with virgin stock, and the waste accumulates in the environment. Between 1950 and 2015, ~80% of plastics produced globally ended up as unmanaged waste [2]. 

The economy of materials and their conversion to products can be defined as circular or linear. The former is articulated within the R-Ladder principle, which concerns the utilization and production of goods within concise cycles defined as R0 Refuse, R1 Rethink, and R2 Reduce. Further intermediate cycles encompass R3 Reuse, R4 Repair, R5 Refurbish, R6 Remanufacture, and R7 Re-purpose, thereby prolonging the lifespan of products. Operating on an extended scale, the long loops are concerned with R8 Recycle and R9 Recover, addressing the end-of-life stage and reframing waste as a resource [10,11]. In contrast, the linear economy dictates that resources are ultimately discarded and either incinerated or sent to landfills [12]. However, the potential loss of materials in a circular economy still needs to be addressed. Should this material loss result in unmanaged waste, it risks becoming an environmental hazard due to the formation of microplastics. This has a negative impact on human health, including cancer and immune system disorders, as well as intestinal, pulmonary, and vascular diseases [13,14,15]. 

To mitigate the impact of persistent microplastics, it is necessary to minimize the instigation of non-degradable waste into the environment and to explore the use of biodegradable alternatives for plastics that end up as unmanaged waste [16]. However, the environmental repercussions of transportation, inadequate quantities of specific materials, and contamination during the utilization phase may undermine the feasibility of recycling efforts [17]. The integration of life cycle assessment (LCA) can provide valuable insights that enable well-informed environmental choices [18]. The life cycle of a product is made up of stages separated by gates. Each stage is described as gate-to-gate, collectively representing the life cycle from beginning to end. Input from the preceding stage is needed to ensure reliable data, but many companies do not record such data, and LCA is, therefore, based on the literature values, assumptions, or simplifications, which reduce the reliability of the results [19]. The LCA tool is listed here for the sake of completion and is not the scope of this research article.

The number of research articles on biopolymers has increased almost exponentially in recent years, but this is not reflected in the volume of marketed products. Between 2016 and 2021, the number of biopolymer-related studies tripled, whereas the market volume increased linearly until 2020 and fell slightly from 2020 to 2021 [2,20]. Biopolymers face significant market entry barriers despite advances that have improved their properties for specific applications. The implementation of such innovations has been slow, reflecting the established infrastructure and supply chain, which is tailored for petrochemical polymers. This article considers the hurdles biopolymers face when entering the market, providing insights to accelerate the adoption of sustainable materials and promote a more circular economy.

### 1.2. Theoretical Framework

**Figure 1:**
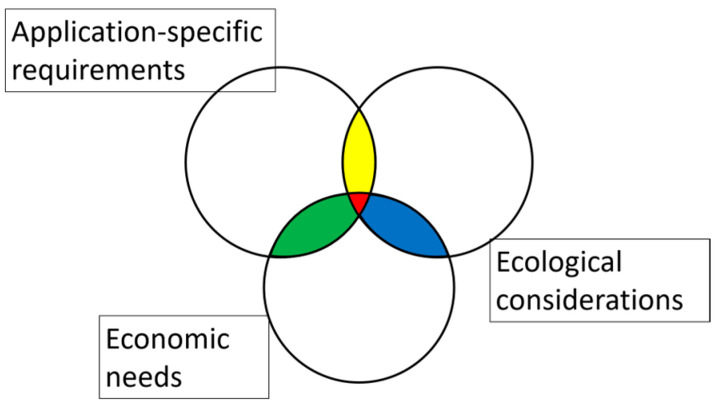
Theoretical framework for the evaluation of barriers that affect the polymers market.

Screening the literature has revealed three essential perspectives that help to explain the challenges described above: application-specific requirements, ecological considerations, and economic needs [21]. Polymer fibers must meet certain requirements, including physical properties, for specific applications before they can be introduced to the market. Ecological considerations include the degradation-by-design approach, which deals with the tailoring of degradability based on the manufacturing process. Several facets of degradation by design are discussed here, based on the comparison of different home-compostable biopolymers produced on the same spinning equipment [22], including the influence of the fiber surface area [23] and the dwell time [19]. However, degradation by design must be tested for economic feasibility. This theoretical framework is depicted in Figure 1.

**Figure 2:**
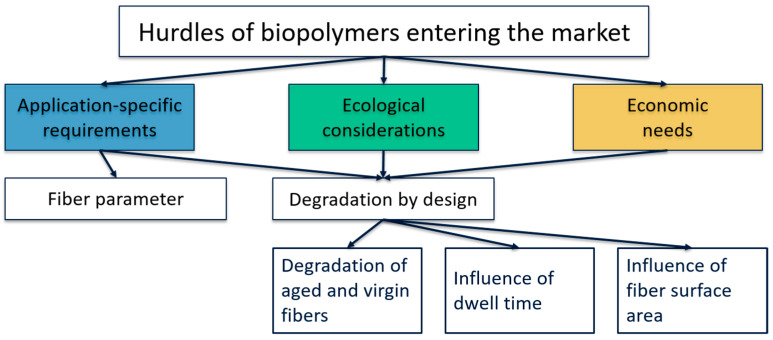
Detailed context of the study design.

The section on application-specific requirements compares the physical properties of common petrochemical polymers and the most frequently used biopolymers. The section on ecological considerations addresses the tailoring of fiber degradation by selecting production parameters such as dwell time and fiber surface area, focusing on the degradation-by-design approach. The section on economic needs assesses the transition to a degradation-by-design approach, providing recommendations for a shift in the product development mindset that emphasizes products created with the understanding that recycling may be less practical, thus increasing the probability of disposal in the environment. This systematic breakdown of the research literature provides a comprehensive understanding, consolidating aspects that are traditionally examined in isolation into a single scholarly inquiry. The context of the study is depicted in more detail in Figure 2. We compare the most widely used non-degradable petrochemical polymers to biodegradable biobased alternatives using six specific examples.

## 2. Application-Specific Requirements

### 2.1. Materials

**Figure 3:**
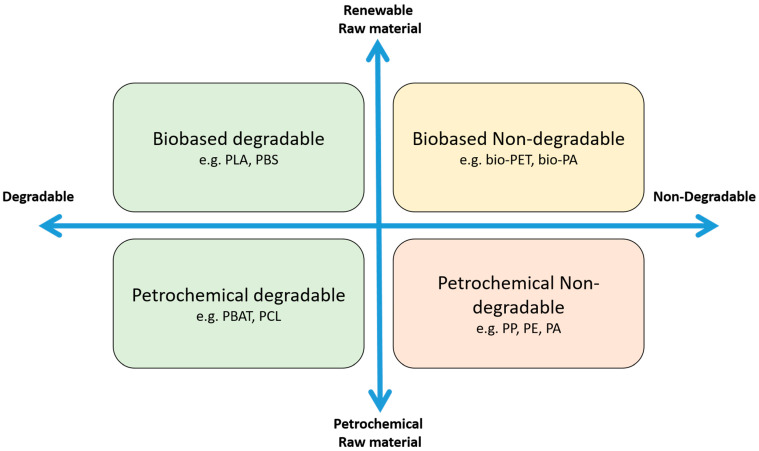
Overview of the four major groups of polymers [27,28,29]. Non-degradable petrochemical polymers are shown in the bottom right, and non-degradable biobased polymers are shown in the top right. Degradable petrochemical polymers are shown in the bottom left side, and degradable biopolymers are shown in the top left. PCL = polycaprolactone, PE = polyethylene. For other abbreviations, see the main text.

When a product is designed, the specifications define what is expected from the product and, ultimately, from the material [24]. Fiber materials can be derived from petrochemical or renewable polymers, which may be degradable or non-degradable (Figure 3). In 2020, petrochemical polymers dominated the market with a total volume of ~109 million metric tons, more than half of which was the polyester polyethylene terephthalate (PET, 52% market share), followed by polyamide (PA, 5.4% market share) and polypropylene (PP, 2.9% market share) [25]. In contrast, the volume of the biopolymer market in the same year was only ~2 million metric tons, led by polybutylene adipate terephthalate (PBAT, 19.2% market share), polylactic acid (PLA, 18.9% market share), and polybutylene succinate (PBS, 3.5% market share) [26]. The market shares for biopolymers are predicted to increase by 2026, reaching 30% for PBAT, 16% for PBS, and 10.4% for PLA [26]. Application-specific requirements of these six polymers are compared below.

### 2.2. Methods

A material cycle achieved by recycling is the optimal outcome for a circular economy because no virgin material is required. However, given that the global recycling rate for polymers is less than 10% [8], a large proportion of material needs to be diverted from landfilling, incineration, or disposal as unmanaged waste, the latter leading to pollution and the creation of persistent microplastics predominantly through mechanical degradation.

For this reason, there is great interest in the degradability of biopolymers [30,31,32]. However, most studies focus on single degradation mechanisms, which only provide a partial explanation for degradation behavior in nature, and this is a significant knowledge gap. Comprehensive research is needed to account for all degradation mechanisms. If a product ends up as unmanaged waste, several degradation mechanisms may occur in parallel or in sequence, including aerobic (in soil) or anaerobic (in water) processes. Initial degradation primarily involves hydrolysis in water combined with UV photolysis, which is followed by biological degradation (microbes and their enzymes).

Biopolymer degradation reduces the polymer chain length, so production parameters that influence this chain length are important. In the spinning process, the temperature, shear stress, residual moisture, and dwell time influence the process-induced degradation of a polymer [33]. Increasing any of these parameters shortens the polymer chains. A balance of parameters is sought for each polymer to achieve a stable process with as little process-induced degradation as possible, but comparisons between different polymers spun on the same machine have only been published recently, examining the influence of dwell time on subsequent degradation [19]. A clear connection was found for PLA, where a longer dwell time induced faster degradation, but the correlation was weaker for PBS. Accordingly, PLA appears to be more process-stable than PBS. The degradation trial was a laboratory-scale disintegration test based on ISO 20200 with virgin and UV-aged samples. PLA was significantly more sensitive than PBS in terms of degradation after processing. The different dwell times were realized using different spinning process scales, which suggests that laboratory and industrial fibers show significant differences.

Given the intricate nature of biopolymer degradation, product degradation must be assessed under conditions that closely mirror natural processes. This approach offers more precise data for the development of effective end-of-life strategies. Fibers were subjected to accelerated weathering (UV and hydrolysis) to simulate aging, with virgin fibers as controls, followed by a disintegration test. The study compared PLA and PBS to PP and considered the effect of dwell time and various fiber cross-sections [19].

### 2.3. Fiber Properties

The properties of PET, PA, and PP (non-degradable petrochemical polymers) are compared to the degradable biopolymers PLA, PBAT, and PBS in Table 1. The fibers show a broad range of properties, including glass transition temperature (T_g_), melting temperature (T_m_), decomposition temperature, tensile strength, elongation at break, and UV resistance. The T_g_ and T_m_ are intrinsically linked to the application field. The three petrochemical polymers are used for different applications due to their distinct characteristics. When these polymers soften during use, they lose their original shape and also their tensile strength. Furthermore, properties such as moisture absorption are essential in applications such as carpets, where a material that absorbs more liquid expands, leading to shrinkage upon drying. Resistance to chemicals, abrasion, and UV exposure is important in many applications. Therefore, all these parameters must be thoroughly assessed and prioritized in an application profile.

Biobased polymers are less well characterized for several parameters, including resistance to chemicals, abrasion, and moisture retention, resulting in data gaps (Table 1). The initial comparison reveals that biopolymers have lower T_g_ and T_m_ values compared to petrochemical polymers. However, the tensile strength of PBAT and PBS are close to PP [34], whereas PLA is closer to PET [35,36,37,38]. The limiting oxygen index (LOI) of PLA is 26, ranking as high as PA. One area where biopolymer alternatives are superior to petrochemical polymers is their degradability under different environmental conditions. PLA is industrially compostable, whereas PBAT and PBS are home compostable. Materials that lose 90% of their mass in compost and break down within 12 months can be described as compostable (depending on national standards). In industrial composting, decomposition occurs at 58 °C, whereas home composting occurs at 28 °C [39].

**Table 1:** Properties of PET, PA 6, PP, PLA, PBAT, and PBS [19,22,40,41,42,43,44,45,46,47,48,49,50,51,52,53,54,55,56,57,58,59]. Empty cells = no values reported in the literature. Performance: ++ = very good, + = good, – = poor.

Properties	Unit	PET		PA 6		PP		PLA	PBAT	PBS
Fiber form		Filament	Spinfibers	Filament	Spinfibers	Filament	Fibers	Fibers	Fibers	Fibers
T_g_	[°C]	80		40–60		–10		55–60	–28	–32
T_m_	[°C]	265		215–220		160–175		160–180	120	110–120
Decomposition temperature	[°C]	300		310–380		300–475		300–372	405	380–415
Shrinkage	[%]	8		0.5–1.5		<3		32		
Density	[g/cm^3^]	1.4		1.14		0.9–0.91		1.25	1.26	1.24–1.28
LOI	[%]	23–25		23–26		17–18		26		
Moisture absorption	[%]	0.2–0.5	0.2–1	3.5–4.5		0				
Water retention capacity	[%]	3–5		10–15						
Tensile strength	[cN/tex]	60–95	30–55	68–85	36–68	15–60	13–60	78.6	11.39	10.62
Elongation at break	[%]	8–20	25–50	14–22	175–310	30–350	20–150	5–7	149.79	136.8
Wet elongation	[%]	100–105		107–150	109–120					
Elastic modulus	[cN/tex]	700–1200	250–400	350–730	175–310	473				
Cross brittleness	[°]	47–48	30–49		35–43					
Chemical resistance		+		+		++				
Abrasion resistance		+		++		+				
UV resistance		+		+		–		–		–
Degradability?		No		No		No		Industrial composting	Home composting	Home composting
Global fiber volume	[t/year]	65,100,000		5,200,000		3,300,000		300,000	200,000	
No. of manufacturers		45		8+		8+		20	2	2
Polymer price (2024)	[€/kg]	0.85		2.1–2.5		1–1.5		1.5–4.5	4–5	4–6

If a recycling rate of less than 10% is assumed, a significant amount of polymer waste is consigned to landfill, incineration, or ends up as unmanaged waste streams. A biopolymer alternative that degrades under environmental conditions reduces the ecological burden of the biopolymer product. If the fiber production volume is compared, PET (65.1 million tons) ranks highest, followed by PA (5.2 million tons) and PP (3.3 million tons). All biopolymer fibers combined account for only 500,000 tons, with PLA ranking first (~300,000 tons). This is reflected by market prices, with petrochemical polymers being the cheapest (PET at 0.85 €/kg is the cheapest overall) and biopolymers being significantly more expensive, with PLA being the most affordable (1.5–4.5 €/kg).

In conclusion, biopolymers can be processed using the same machinery and settings as petrochemical polymers, resulting in fibers with comparable technical properties, suggesting that replacement is possible. However, the lack of data (empty cells in Table 1) indicates more research is needed. The low market volume (due to fewer biopolymer manufacturers [44]) and higher market prices are also major barriers. Competition among polymer manufacturers may lead to price reductions, making biopolymers a more appealing option for processing companies in terms of cost and dependency.

### 2.4. Challenges Based on Application-Specific Requirements

The successful introduction of a new material onto the market is intricately linked to the availability of comprehensive data. The performance of the new material should closely match that of current materials with similar applications. Based on comparable physical parameters, such as tensile strength, LOI, and T_g_, the three biopolymers discussed above have properties that make them suitable for applications currently utilizing PP [34,35,36], whereas only PLA is a suitable replacement for PET [35,36]. The paucity of data remains a significant hurdle.

Another aspect that must be considered is availability. Biopolymers have a smaller market share, with fewer manufacturers to supply the market [44]. The overall cost of biopolymers is therefore higher, but the added benefits of switching from oil to renewable resources and the degradability of the resulting products mean that drop-in polymers can reduce pollution and eliminate the costs of environmental clean-up to balance the overall costs throughout the lifecycle [60]. The cost of new spinning equipment should also be considered, so biopolymers that can be spun on conventional spinning equipment are advantageous. The comparison of home-compostable biopolymers on the same machine with the same settings has proven that this is possible [22]. 

In conclusion, biopolymers can be processed using the same machinery and settings as petrochemical polymers, resulting in fibers with comparable technical properties. However, the limited market volume, the small number of polymer manufacturers, the higher cost, and the lack of comprehensive data pose significant barriers to the widespread adoption of biopolymers. Increased competition among polymer manufacturers may lead to price reductions that make biopolymers more appealing for processing companies in terms of cost and dependency.

## 3. Ecological Considerations

### 3.1. The Ecological Importance of Polymer Degradation

The degradation of polymers is determined not only by their inherent properties but also by external factors, such as temperature, the presence of microorganisms, pH, humidity [61], and other climatic variables. Degradation only takes place if a temperature threshold is reached, which is specific for every polymer and related to the T_g_ [62,63]. This allows the design of polymers that remain stable until they reach a given temperature. The absence of microorganisms, oxygen, or humidity reduces the degradation rate. Consequently, the rate of degradation varies across different geographical locations. Even so, these diverse environmental conditions can be factored into product design, thereby enabling targeted degradation by leveraging the production process (such as spinning) to modulate the behavior of biodegradable polymers.

Polymer degradation is defined as a surface process [64]. The larger the surface area (assuming identical chain lengths and crystallinity) in relation to its cross-sectional area, a parameter known as the circumference/cross-sectional relationship (CCR), the more rapidly fibers break down. This was confirmed by comparing PLA and PBS fibers with different cross-sections, showing that a larger CCR accelerates degradation [23]. However, a fiber sample with a smaller surface-to-mass ratio can break down completely when aged with UV, whereas a corresponding virgin sample is not completely degraded. The literature makes no distinction between virgin and aged samples. Many studies examine virgin laboratory fibers in a single degradation test, which does not reflect real processes in the environment. Natural degradation involves the initial hydrolysis and UV photolysis of industrial fibers, followed by biological degradation in soil or water. Therefore, polymer tests based on laboratory spinning and subsequent degradation via a single mechanism must be fundamentally reconsidered.

To investigate the difference between laboratory and industrially spun fibers, the degradation of PLA and PBS fibers was compared with different dwell times and with both virgin fibers and those subject to accelerated weathering (UV and water). A longer dwell time prompted faster degradation, especially for PLA, which was completely degraded [19]. These insights make it possible to control the degradation of PLA and PBS fibers to a certain extent based on the fiber surface properties and dwell time during spinning. The production of tailored degradable fibers is also facilitated by the ability to process biopolymers on the same spinning equipment as petrochemical polymers [22]. By understanding the relationship between process parameters and degradation properties, application-specific degradation profiles can be created for individual fibers and used in product design.

### 3.2. Challenges Based on Ecological Considerations

Biopolymer degradation in the literature is predominantly examined under controlled laboratory conditions, typically with single mechanisms such as UV exposure or degradation tests in soil. Simultaneous or consecutive mechanisms are rarely considered. However, when polymer products enter the environment, they are subjected to multiple degradation mechanisms that act together or in sequence and may show dependencies, such as initial UV photolysis caused by sunlight and hydrolysis caused by immersion or exposure to rain, prior to degradation in soil [19,23]. It is, therefore, essential to consider this sequence of events to accurately predict the degradation of biopolymer products under real-world conditions. The degradation-by-design approach offers a significant opportunity to enhance the product design process. Where a product is unlikely to be recyclable and may be consigned to landfill or incineration or end up as an unmanaged waste stream, degradability can be a game-changer with immense environmental benefits. The ability to compost products locally reduces the volume of waste and logistical challenges [16,17]. Degradation by design provides a means to adjust degradability to suit different applications, offering advantages that can remove current market barriers to biopolymers. 

## 4. Economic Needs

### 4.1. The Economic Aspects of Petrochemical and Biobased Polymers

The literature review for this article revealed agreement between industry experts and scholars that a comprehensive approach is needed to address the use of degradable, biobased plastics. Consumers claim such an approach is lacking and that the industry should adopt more ecological processes, but they still practice price-sensitive decision-making regarding functional comparability [65,66,67]. Manufacturing companies also try to reduce costs while sourcing production materials [68].

Despite the potential impact of degradation by design on business models and profitability, companies often overlook this approach to stay competitive [69,70]. This behavior, rooted in resource-based academic views and business models, follows the theory of constraints, which is often considered a baseline for allocating tangible and intangible resources to gain competitive advantages. Examples of these resources include capabilities, processes, knowledge, and capital [71]. However, some academic scholars have used the concepts of exploitation and exploration [72] to describe the capability of an organization to manage the conflicting allocation of scarce resources to existing capabilities or the development of new ones. We look at degradation by design from a multidisciplinary perspective, supported by researchers who see no conflict between these economic and ecological drivers but rather an opportunity to develop new business models that foster profitability in a sustainable manner [73,74].

### 4.2. Challenges Based on Current Economic Models

From an economic perspective, we must consider factors such as market volume, the number of manufacturers, and raw material prices. In the context of textile applications, the selection of materials is intricately tied to their availability and costs [67]. Manufacturers play a significant role in the limited availability and higher prices of biopolymers, particularly PBS and PBAT. The degradation-by-design approach is an opportunity to re-evaluate the fundamental product design approach, helping to overcome challenges caused by the significantly higher price of biopolymer raw materials. 

## 5. Degradation by Design in a Circular Economy

### 5.1. The Potential Benefits of Degradation by Design

Although the recycling of polymer materials is the preferential end-of-use scenario, this may be impossible in many cases due to contamination (or the manufacture of composites) or logistical constraints [17]. In these cases, degradation is a highly beneficial end-of-life alternative for managed waste streams and is desirable for unmanaged waste streams to avoid pollution. This strategic approach will reduce the accumulation of non-degradable polymers in the environment, ultimately mitigating the harm caused by persistent microplastics. A quantitative LCA can evaluate suitable materials, manufacturing processes, and end-of-life options.

**Figure 4:**
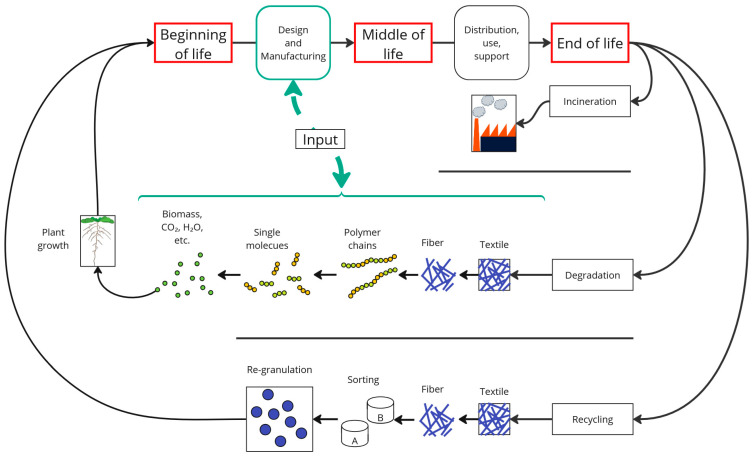
Possible end-of-life strategies for single-use textiles.

An in-depth understanding of product development can be acquired by delving into different methods that provide alternative perspectives on the product life cycle (Figure 4). For example, a visual representation of the product life cycle can outline the journey from raw materials to production and recycling, with waste generated at different stages carefully managed by recycling, whereas non-recyclable waste is directed to landfill or incineration [75]. Conversely, a more linear approach illustrates the unidirectional flow of goods and information with limited interaction between end-of-life considerations and design aspects, which can be addressed by integrating retailer information to inform the design process [76].

Composting, particularly of non-recyclable textiles contaminated with organic matter, is not considered in these methods and requires more research [77]. Factoring in the likelihood of improper disposal makes it necessary to consider degradation as a means to reduce the buildup of plastic waste. Additionally, the resulting compost can be used as a fertilizer at the beginning of the raw material production process for biobased polymers, thereby ensuring a supply of new raw materials. If recycling is impossible, particularly for textiles contaminated with organic material, incineration is currently the typical disposal route. But for textiles consigned to landfills or discarded in the environment, degradable biopolymers provide a beneficial end-of-life scenario. If this is already considered during product design, this alternative to incineration and recycling would reduce the accumulation of non-degradable landfill waste and also allow the disposal of products by home composting.

### 5.2. Challenges Triggered by the Degradation-by-Design Approach

The environmental repercussions of polymers have become a matter of considerable significance to the public, and the entire product lifecycle (including its post-use and disposal phases) must be taken into account during development. Degradation by design is therefore not just a strategy but a responsibility for society to address the escalating problem of recalcitrant microplastics in the environment. Integrating biobased, degradable polymers into product design can reduce the likelihood of such materials permeating natural ecosystems. This approach effectively tackles the end-of-life stage of products and is aligned with the fundamental principles of sustainability and environmental stewardship. In essence, by proactively contemplating a product’s environmental impact and engineering it to degrade responsibly, substantial progress can be made to mitigate the challenge of resistant microplastics in our delicate ecosystems. 

## 6. Discussion

The mechanical properties of petrochemical polymers such as PA often outperform those of biopolymers. This is due to the controlled polymerization processes, which yield linear and faceted structures with high cohesion and stability. However, biopolymers such as PLA, PBS, and PBAT can achieve comparable mechanical strength (Table 1). Thermal properties are also important. Biopolymers generally have lower thermal application ranges than their petrochemical counterparts. For example, PP has a higher Tm (160–170 °C) than PLA (150–160 °C) and a significantly higher T_m_ than PBS (115 °C).

The T_g_ of PLA is in the 55–60 °C range, whereas that of PBS is –30 °C. The tenacities of PLA, PBS [34,35,36], and PBAT are, therefore, similar to PP. The tenacity of PLA is comparable to PET [35,36]. The T_m_ and T_g_ of PBAT and PBS are close to PP, whereas PLA ranks between PA and PET (Table 1). However, biopolymers are more difficult to process and more susceptible to process-related degradation than petrochemical polymers [19,33]. 

Although the higher price of biopolymers, lower global production volumes, and relatively small number of manufacturers currently limit their market penetration (Table 1) [34,36,57,58,59,67,78], this is being addressed by ongoing research into their diverse applications. Despite the challenge of material selection for product design, the potential to reduce costs provides opportunities to overcome these hurdles. The physical comparability of PBAT and PBS to PP and PLA to PET, at least for some parameters, suggests that biopolymers can be dropped where PP and PET are used. 

Future research avenues include the testing of biobased polymer fibers to close the gaps in knowledge evident in Table 1. More comprehensive research will broaden the applications of biopolymers because companies can predict the suitability of biopolymers for new applications. The degradation-by-design approach should also be studied in more detail, expanding beyond characteristics such as fiber surface area and residence time in the spinning device. Other fiber properties and process parameters should be investigated, and a wider range of biopolymers should be considered, leading to a database that could later give rise to a software-based optimization tool for the design of an optimal spinning process for specified degradation behaviors.

## 7. Conclusions

The research summarized in this article suggests there is no need for investment in new equipment because biopolymers can be spun on existing machines using the same parameters. The operating temperatures of these biopolymers are also very close to those of petrochemical polymers. However, the lack of data on biopolymers in the literature poses a significant obstacle because not every application is solely based on tenacity, T_g_, and T_m_. Table 1 also highlights the higher market volume and number of manufacturers of petrochemical polymers, leading to far lower prices. Given the comparable physical properties of biopolymers and petrochemical polymers, the primary challenges appear to be related to availability and price.

The comparison of laboratory and real-world degradation reveals differences in ecological impact. When only a single degradation mechanism is examined, the observed degradation behavior significantly diverges from a pre-exposure scenario. This underscores the need to reconsider the evaluation of biopolymer degradation by introducing more accurate simulations of real-world conditions [19,23]. Expanding on this, the concept of degradation by design considers suitability for composting at the outset of product design. Degradation behavior can be tailored by modifying process parameters to better align with real-world degradation scenarios. This concept has been presented mainly in product design, focusing on product recyclability. But for products that cannot be recycled and those ending up in unmanaged waste streams, degradation offers the potential to mitigate the formation of persistent microplastics, as well as a significant decrease in waste volume and transportation needs.

## Data Availability

The datasets used and/or analyzed during this study are available from the corresponding author upon reasonable request.
